# Supplementary material for: Modulated Electro-Hyperthermic (mEHT) Treatment in the Therapy of Inoperable Pancreatic Cancer Patients—A Single-Center Case-Control Study
Source: Diseases. 2021 Nov 3;9(4):81. doi: 10.3390/diseases9040081 (PMC8628793; doi:10.3390/diseases9040081)
Supplement: Supplementary file 1 [file diseases-09-00081-s001.zip › diseases-1343334-supplementary.pdf]

Table S1. Basic demographic and clinical characteristics are summarized of the case and control groups (mean ± SD (median and (range)), which were further subdivided by the presence of metastasis. Unit of frequency data is the number of observations (percentage).

Parameter	mEHT treated, without metastasis (n = 19)	mEHT treated, with metastasis (n = 20)	Control, without metastasis (n = 15)	Control, with metastasis (n = 24)	*p*-Value
Age (years)	66.75 ± 11.96 (69 (45 – 82))	65.09 ± 7.69 (65 (48 – 85))	67.06 ± 9.42 (67 (47 – 78))	65.37 ± 8.42 (66 (46 – 76))	Case-Control: 0.9526 [1] Metastasis: 0.4381 [1]
Male gender	10 (52.6)	8 (44.0)	5 (33.3)	13 (54.2)	0.9740 [2]
Location of the tumor					
- Head of the pancreas	11 (57.9)	9 (45)	10 (66.7)	16 (66.6)	0.3159 [2]
- Body of the pancreas	7(36.8)	7 (35)	4 (26.6)	4 (16.7)	
- Tail of the pancreas	1 (5.3)	4 (20)	1 (6.7)	4 (16.7)	
Without radiologically detected ascites	13 (61.9)	8 (38.1)	10 (38.5)	16 (61.5)	0.2006 [2]
Overall survival (month)	19.83 ± 10.26 (19.78 (6.5 – 47.1))	14.23 ± 6.02 (14.82 (4.4 – 23.0))	18.54 ± 14.44 (11.6 (7.0 – 48.8))	11.48 ± 6.93 (10.5 (2.4 – 31.8))	Case-Control: 0.1979 [1] Metastasis: 0.0045 [1]
Progression-free survival (month)	14.20 ± 8.72 (12.91 (4.5 – 34.8))	9.65 ± 4.09 (9.38 (4.2 – 18.2))	10.44 ± 5.06 (8.87 (3.4 – 26.0))	7.33 ± 5.31 (5.36 (1.6 – 18.4))	Case-Control: 0.0164 [1] Metastasis: 0.0067 [1]
1-year survival	14 (73.7)	12 (60.0)	6 (40.0)	10 (41.6)	0.0290 [2]
2-year survival [#]	6 (31.6)	0 (0.0)	4 (26.6)	1 (4.2)	0.9420 [2]
3-year survival [#]	1 (5.3)	0 (0.0)	3 (20.0)	0 (0.0)	0.2303 [2]
1-year PFS	10 (52.6)	5 (25.0)	2 (13.3)	5 (20.8)	0.0587 [2]
2-year PFS	2 (10.5)	0 (0.0)	1 (6.7)	0 (0.0)	0.6979 [2]
Number of mEHT treatments	62.53 ± 17.54 (65 (32-102))	56.00 ± 32.80 (52 (21 – 154))	–	–	0.3321 [3]
Time to first mEHT treatment (days)	105.74 ± 171.30 (46 (73 – 718))	93.70 ± 125.94 (44 (6 – 445))	–	–	0.8993 [3]
OS after the first mEHT treatment (month)	16.35 ± 7.06 (17.6 (5.0 – 22.7))	11.15 ± 6.33 (9.3 (2.3 – 21.4))	–	–	0.0192 [3]
OS after the last mEHT treatment (month)	8.22 ± 6.37 (8.2 (0.3 – 20.6))	5.01 ± 4.93 (8.2 (0.3 – 16.7))	–	–	0.2110 [3]
PFS after the first mEHT treatment (month)	10.72 ± 6.28 (10.7 (3.0 – 23.0))	6.57 ± 3.63 (6.2 (1.8 – 16.6))	–	–	0.0305 [3]
PFS after the last mEHT treatment (month)	2.59 ± 6.24 (1.8 (-9.0 – 15.8))	0.43 ± 2.67 (0.61 (-7.2 – 6.1))	–	–	0.3761 [3]

[1] Two-way ANOVA, [2] Cochran-Mantel-Haenszel test, [3] Wilcoxon-Mann-Whitney rank sum test. mEHT: modulated electro-hyperthermia, OS: overall survival, PFS: progression-free survival. [#] 2 and 4 right-censored case patients had lower observation time than 24 and 36 months, respectively, and 1 censored control patient had a censor time between 24 – 36 months.

Table S2. Basic demographic and clinical characteristics are summarized of the case and control groups (mean ± SD (median and (range)), which were further subdivided by the presence of ascites. Unit of frequency data is the number of observations (percentage).

Parameter	mEHT treated, without ascites (n = 21)	mEHT treated, with ascites (n = 18)	Control, without ascites (n = 26)	Control, with ascites (n = 13)	*p*-Value
Age (years)	67.08 ± 10.69 (69 (45 – 84))	64.51 ± 8.99 (65 (46 – 81))	66.63 ± 9.83 (69 (45 – 78))	64.81 ± 6.15 (64 (51 – 76))	Case-Control: 0.9525 [1] Metastasis: 0.3135 [1]
Male gender	11 (52.4)	7 (38.9)	11 (42.3)	7 (53.8)	0.9851 [2]
Location of the tumor					
- Head of the pancreas	13 (61.9)	7 (38.9)	17 (65.4)	9 (69.2)	0.3506 [2]
- Body of the pancreas	1 (4.8)	4 (22.2)	4 (15.4)	1 (7.7)	
- Tail of the pancreas	7 (33.3)	7 (38.9)	52 (19.2)	3 (23.1)	
Without distant metastasis	13 (61.9)	6 (33.3)	10 (38.5)	5 (38.5)	0.2838 [2]
Overall survival (month)	19.35 ± 8.94 (21.26 (6.5 – 47.1))	11.16 ± 7.78 (12.76 (4.4 – 34.8))	14.79 ± 10.53 (10.30 (4.1 – 45.4))	13.01 ± 11.85 (11.04 (2.4 – 48.8))	Case-Control: 0.2149 [1] Metastasis: 0.1202 [1]
Progression-free survival (month)	12.46 ± 6.96 (10.74 (4.5 – 32.4))	11.17 ± 7.29 (10.02 (4.2 – 34.8))	9.35 ± 5.77 (8.84 (1.6 – 26.0))	6.88 ± 4.19 (6.93 (2.1 – 16.4))	Case-Control: 0.0208 [1] Metastasis: 0.2090 [1]
1-year OS	16 (76.2)	10 (55.6)	11 (42.3)	5 (38.5)	0.0176 [2]
2-year OS [#]	4 (19.0)	2 (11.1)	4 (15.4)	1 (7.7)	0.5636 [2]
3-year OS [#]	1 (4.7)	0 (0.0)	2 (7.7)	1 (7.7)	0.4100 [2]
1-year PFS	9 (42.9)	6 (33.3)	5 (19.2)	2 (15.4)	0.0398 [2]
2-year PFS	1 (4.7)	1 (5.6)	1 (3.8)	0 (0.0)	0.5375 [2]
Number of mEHT treatments	66.86 ± 28.16 (67 (22 – 154))	50.22 ± 21.12 (68 (37 – 85))	–	–	0.0648 [3]
Time to first mEHT treatment (days)	82.90 ± 151.88 (37 (2 – 718))	119.00 ± 144.91 (133 (6 – 445))	–	–	0.4301 [3]
OS after the first mEHT treatment (month)	16.63 ± 6.22 (17.94 (5.0 – 26.7))	10.25 ± 6.67 (8.43 (2.3 – 26.6))	–	–	0.0028 [3]
OS after the last mEHT treatment (month)	8.64 ± 6.18 (9.50 (0.3 – 20.6))	4.16 ± 4.44 (2.38 (0.3 – 15.8))	–	–	0.0322 [3]
PFS after the first mEHT treatment (month)	9.74 ± 5.40 (8.80 (2.4 – 23.0))	7.26 ± 5.35 (5.80 (1.8 – 22.7))	–	–	0.0883 [3]
PFS after the last mEHT treatment (month)	1.75 ± 5.03 (0.95 (-7.2 – 12.4))	1.17 ± 4.82 (0.49 (-9.0 – 15.8))	–	–	0.7142 [3]

[1] Two-way ANOVA, [2] Cochran-Mantel-Haenszel test, [3] Wilcoxon-Mann-Whitney rank sum test. mEHT: modulated electro-hyperthermia, OS: overall survival, PFS: progression-free survival. [#] 2 and 4 right-censored case patients had lower observation time than 24 and 36 months, respectively, and 1 censored control patient had a censor time between 24 – 36 months.

Table S3a. Basic demographic and clinical characteristics are summarized of the case and control groups (mean ± SD (median and (range)), which were further subdivided by the presence of ascites and/or metastasis. Unit of frequency data is the number of observations (percentage).

Parameter	mEHT treated, without ascites without metastasis (n = 13)	mEHT treated, with ascites without metastasis (n = 6)	mEHT treated, without ascites with metastasis (n = 8)	mEHT treated, with ascites with metastasis (n = 12)	*p*-Value
Age (years)	67.79 ± 11.88 (70 (45 – 82))	64.50 ± 12.93 (68 (46 – 81))	65.93 ± 9.07 (65 (56 – 85))	64.52 ± 6.70 (65 (48 – 76))	Case-Control: 0.9530 [1] Metastasis: 0.6882 [1]
Male gender	7 (53.8)	3 (50.0)	4 (50.0)	4 (33.3)	0.8160 [2]
Location of the tumor					
- Head of the pancreas	7 (53.8)	4 (66.7)	6 (75.0)	3 (25.0)	0.4629 [2]
- Body of the pancreas	1 (7.7)	0 (0.0)	0 (0.0)	4 (33.3)	
- Tail of the pancreas	5 (38.5)	2 (33.3)	2 (25.0)	5 (41.7)	
Overall survival (month)	20.47 ± 10.59 (21.32 (6.5 – 47.1))	18.43 ± 10.29 (13.55 (10.2 – 34.8))	17.53 ± 5.44 (10.28 (10.1 – 23.0))	12.03 ± 5.52 (11.37 (4.4-18.3))	Case-Control: 0.1985 [1] Metastasis: 0.0206 [1]
Progression-free survival (month)	14.75 ± 7.87 (14.85 (4.5 – 32.4))	13.00 ± 11.08 (9.05 (5.0 – 34.8))	8.74 ± 2.61 (8.23 (6.0 – 13.7))	10.26 ± 4.86 (10.02 (4.2 – 18.2))	Case-Control: 0.0172 [1] Metastasis: 0.0410 [1]
1-year OS	10 (76.9)	4 (66.7)	6 (75.0)	6 (50.0)	0.0202 [2]
2-year OS [#]	4 (30.8)	2 (33.3)	0 (0.0)	0 (0.0)	0.8662 [2]
3-year OS [#]	1 (7.7)	0 (0.0)	0 (0.0)	0 (0.0)	0.2392 [2]
1-year PFS	8 (61.5)	2 (33.3)	1 (12.5)	4 (33.3)	0.0664 [2]
2-year PFS	1 (7.7)	1 (16.7)	0 (0.0)	0 (0.0)	0.7019 [2]
Number of mEHT treatments	64.69 ± 18.3 (65 (32 – 102))	57.83 ± 16.29 (59 (40 – 80))	70.38 ± 40.84 (69 (22 – 154))	46.42 ± 22.84 (47 (21 – 85))	0.1799 [3]
Time to first mEHT treatment (days)	102.38 ± 190.49 (46 (2– 718))	113.00 ± 135.88 (56 (15-371))	51.25 ± 43.09 (33 (14-130))	122.00 ± 155.01 (50 (6-445))	0.3753 [3]
OS after the first mEHT treatment (month)	17.11 ± 6.82 (17.94 (5.0-26.7))	14.72 ± 7.95 (10.99 (8.1-26.6))	15.84 ± 5.44 (17.87 (9.1-21.4))	8.02 ± 4.87 (6.92 (2.3-18.0))	0.0052 [3]
OS after the last mEHT treatment (month)	8.79 ± 6.55 (10.87 (0.3 – 20.6))	7.00 ± 6.34 (4.80 (1-15.8))	8.40 ± 5.94 (6.95 (1.6-16.7))	2.74 ± 2.38 (1.82 (0.3 – 8.4))	0.0415 [3]
PFS after the first mEHT treatment (month)	11.39 ± 5.96 (12.62 (3.0 – 23.0))	9.29 ± 7.29 (6.09 (13.9-22.6))	7.05 ± 3.02 (6.46 (2.4-12.1))	6.25 ± 4.09 (4.98 (1.8-16.6))	0.0896 [3]
PFS after the last mEHT treatment (month) *	3.07 ± 5.34 (3.0 (-5.8-12.4))	1.57 ± 8.63 (-0.94 (-9.0 – 15.8))	-0.39 ± 3.88 (0.26 (-7.2 – 6.1))	0.97 ± 1.4 (0.85 (-0.9 – 3.7))	0.4577 [3]

[1] Two-way ANOVA, [2] Cochran-Mantel-Haenszel test, [3] One-way ANOVA. mEHT: modulated electro-hyperthermia, OS: overall survival, PFS: progression-free survival. Note: *p*-values are the same in Table S3a and S3b as comparison was performed with all 8 subgroups in a "2x4" manner. [#] 2 and 4 right-censored case patients had lower observation time than 24 and 36 months, respectively, and 1 censored control patient had a censor time between 24 – 36 months. * Negative values in 'PFS after the last mEHT treatment' means that progression developed during the mEHT treatments.

Table S3b. Basic demographic and clinical characteristics are summarized of the case and control groups (mean ± SD (median and (range)), which were further subdivided by the presence of ascites and/or metastasis. Unit of frequency data is the number of observations (percentage).

Parameter	Control, without ascites without metastasis (n = 10)	Control, with ascites without metastasis (n = 5)	Control, without ascites with metastasis (n = 16)	Control, with ascites with metastasis (n = 8)	*p*-Value
Age (years)	67.73 ± 9.90 (68 (47 – 78))	65.72 ± 9.31 (67 (51 – 76))	65.94 ± 10.04 (70 (46 – 76))	64.25 ± 3.80 (64 (59 – 780))	Case-Control: 0.9530 [1] Metastasis: 0.6882 [1]
Male gender	2 (20.0)	3 (60.0)	98 (56.3)	4 (50.0)	0.8160 [2]
Location of the tumor					
- Head of the pancreas	7 (70.0)	3 (60.0)	10 (62.5)	6 (75.0)	0.4629 [2]
- Body of the pancreas	0 (0.0)	1 (20.0)	4 (25.0)	0 (0.0)	
- Tail of the pancreas	3 (30.0)	1 (20.0)	2 (12.5(	2 (25.0)	
Overall survival (month)	18.54 ± 13.86 (10.92 (8.4 – 45.4))	18.56 ± 17.25 (11.76 (7.0 – 48.8))	12.44 ± 7.37 (10.28 (4.1-31.8))	9.54 ± 5.92 (10.81 (2.4 – 19.0))	Case-Control: 0.1985 [1] Metastasis: 0.0206 [1]
Progression-free survival (month)	11.20 ± 5.32 (10.05 (8.3-26.0))	8.94 ± 4.67 (8.25 (3.4-16.4))	8.20 ± 5.91 (6.60 (1.6 – 18.4))	5.60 ± 3.57 (4.43 (2.1 – 12.8))	Case-Control: 0.0172 [1] Metastasis: 0.0410 [1]
1-year OS	4 (40.0)	2 (40.0)	7 (43.8)	3 (37.5)	0.0202 [2]
2-year OS [#]	3 (30.0)	1 (20.0)	1 (6.3)	0 (0.0)	0.9591 [2]
3-year OS [#]	2 (20.0)	1 (20.0)	0 (0.0)	0 (0.0)	0.1952 [2]
1-year PFS	1 (10.0)	1 (20.0)	4 (25.0)	1 (12.5)	0.0664 [2]
2-year PFS	1 (10.0)	0 (0.0)	0 (0.0)	0 (0.0)	0.7019 [2]

[1] Two-way ANOVA, [2] Cochran-Mantel-Haenszel test. Note: *p*-values are the same in Table S3a and S3b as comparison was performed with all 8 subgroups in a "2x4" manner. [#] 2 and 4 right-censored case patients had lower observation time than 24 and 36 months, respectively, and 1 censored control patient had a censor time between 24 – 36 months.

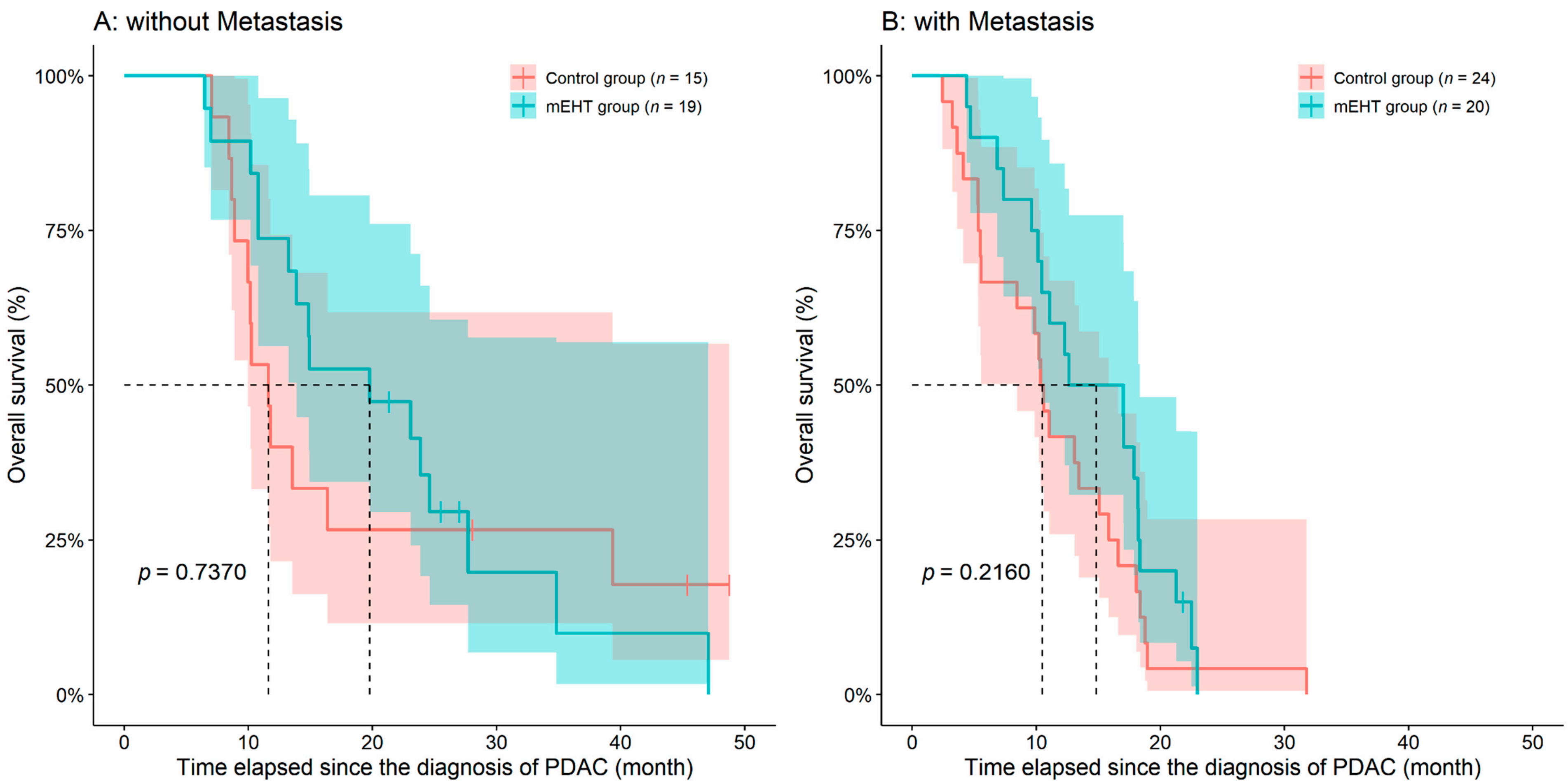

Figure S1. Inoperable pancreatic ductal adenocarcinoma (PDAC) patients treated with adjuvant modulated electro-hyperthermia (mEHT) had a trend to better survival both with (**B**) and without (**A**) the presence of metastases. The dotted line and the lighter colored intervals represent median survival and the asymmetrical 95% confidence interval, respectively.

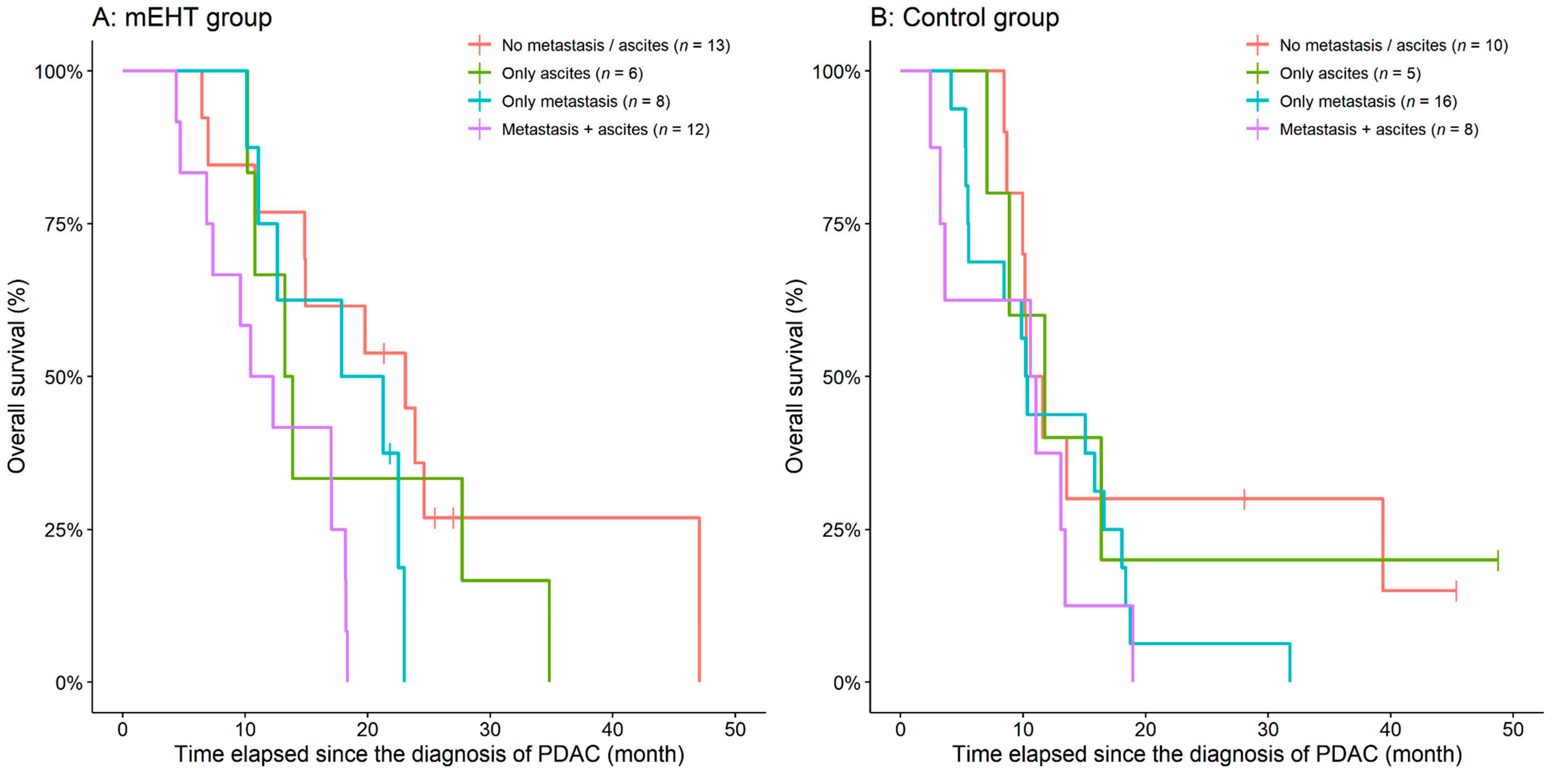

Figure S2. Differences in overall survival of inoperable pancreatic ductal adenocarcinoma (PDAC) patients who have been treated with (**A**) or without (**B**) adjuvant modulated electro-hyperthermia (mEHT) if ascites and/or metastases emerge.

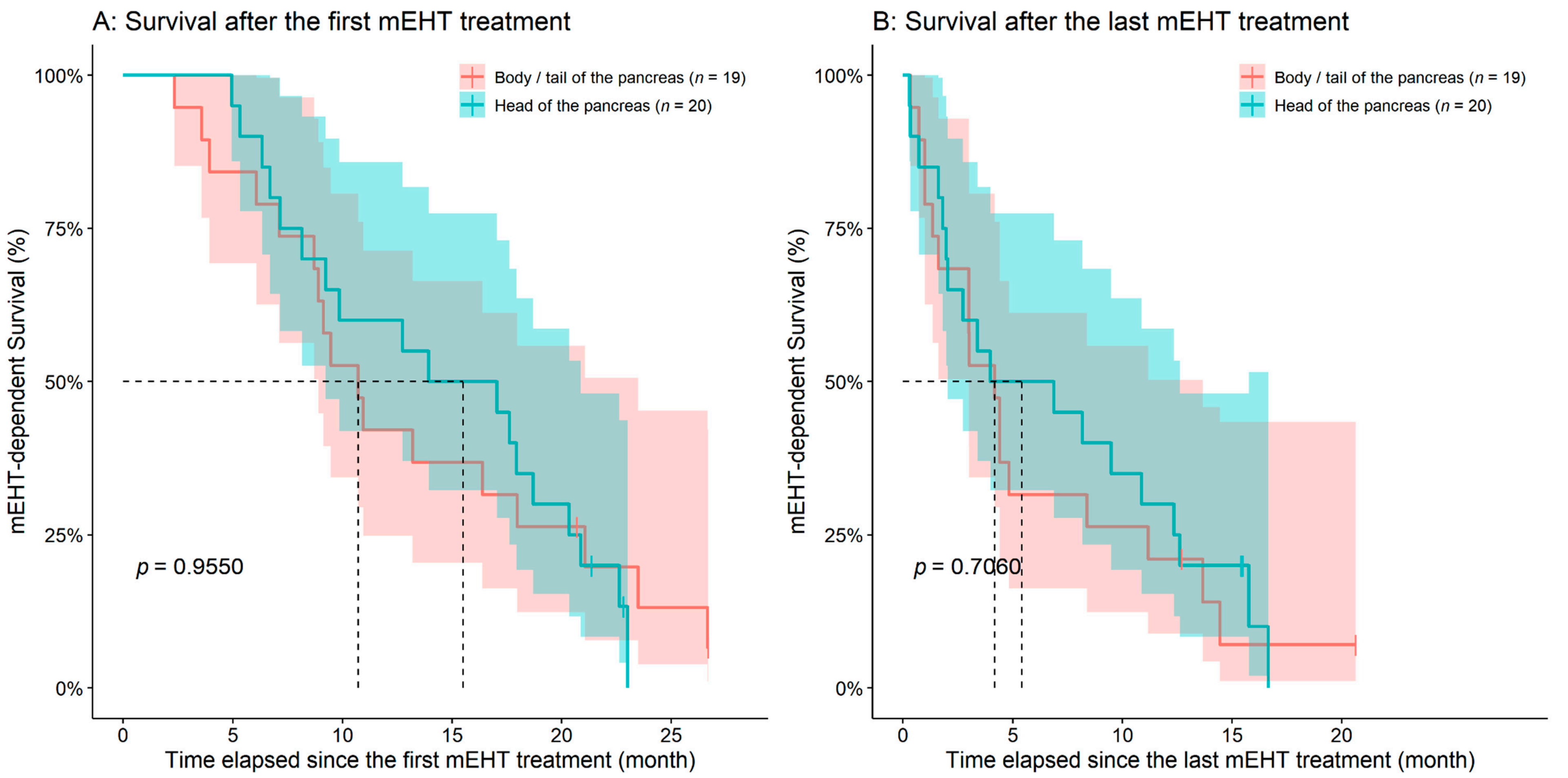

Figure S3. The location of the tumor on the pancreas did not affected the survival times after both the first (**A**) and the last (**B**) modulated electro-hyperthermia (mEHT) treatment. The dotted line and the lighter colored intervals represent median survival and the asymmetrical 95% confidence interval, respectively.
